# High-dose methylprednisolone pulse therapy for treatment of refractory intestinal involvement caused by Henoch–Schönlein purpura: a case report

**DOI:** 10.1186/s13256-015-0545-4

**Published:** 2015-03-24

**Authors:** Hyun Sik Kang, Hee Sup Chung, Ki-Soo Kang, Kyoung Hee Han

**Affiliations:** 1 Department of Pediatrics, Jeju National University Hospital, Aran 13gil 15, Jeju-si, Jeju Special Self-Governing Province 690-767 Korea; 2 Jeju National University School of Medicine, Aran 13gil 15, Jeju-si, Jeju Special Self-Governing Province 690-767 Korea; 3 Department of Pediatrics, Jeju National University School of Medicine, Aran 13gil 15, Jeju-si, Jeju Special Self-Governing Province 690-767 Korea

**Keywords:** Duodenitis, Gastrointestinal tract, Henoch–Schönlein purpura, Methylprednisolone, Pulse therapy

## Abstract

**Introduction:**

Henoch–Schönlein purpura is an immunoglobulin A-mediated, small vascular inflammatory disease that can be associated with palpable purpura, arthralgia, abdominal pain, or nephritis. The presence of purpura facilitates the diagnosis of Henoch–Schönlein purpura at the onset of associated symptoms, whereas the absence of purpura makes the diagnosis challenging. It is important to diagnose Henoch–Schönlein purpura with delayed-onset skin purpura to avoid unnecessary surgery for acute abdomen. Most cases of Henoch–Schönlein purpura with severe abdominal pain are treated with low-dose steroids and intravenous immunoglobulin.

**Case presentation:**

A 15-year-old Korean girl complained of severe abdominal pain and delayed-onset purpura on admission. Henoch–Schönlein purpura was diagnosed based on endoscopic findings of hemorrhagic duodenitis and duodenal vasculitis and abdominal computed tomography findings of edematous bowels. Two common initial treatments, a low-dose steroid and intravenous immunoglobulin, were administered, but there was no improvement for 1 month. Subsequently, we used high-dose intravenous methylprednisolone pulse therapy (30mg/kg/day, with a maximum of 1g/day), which dramatically alleviated her abdominal symptoms.

**Conclusions:**

High-dose intravenous methylprednisolone pulse therapy can be used as the ultimate treatment for delayed-onset Henoch–Schönlein purpura with severe abdominal pain when symptoms do not improve after low-dose steroid and intravenous immunoglobulin treatments.

## Introduction

Henoch–Schönlein purpura (HSP) is one of the most common small vascular inflammatory diseases in childhood that is associated with an immunoglobulin (Ig) A-mediated autoimmune response [1]. The main clinical symptoms of HSP, including purpura, arthralgia, abdominal pain, and nephritis [2], which are associated with vascular injuries of the skin, joint areas, abdomen, and kidneys, do not always occur simultaneously [3]. HSP can be characterized by palpable purpura mostly over the buttocks and lower extremities. Compared to other clinical manifestations, palpable purpura is present in all patients with HSP [2]. HSP can be diagnosed if palpable purpura is present with diffuse abdominal pain, any biopsy showing predominant IgA deposition, arthritis, arthralgia, or renal involvement [2]. Therefore, it may be difficult to diagnose HSP based only on associated symptoms if palpable purpura is momentarily absent.

Intravenous Ig (IVIg) and low-dose steroids have been used for treating HSP with gastrointestinal (GI) involvement [4,5]. When HSP with GI involvement is diagnosed, a low-dose steroid (1 to 2mg/kg/day) can be administered initially, followed by IVIg if the symptoms persist [4]. When purpura is present, the response to these treatments is favorable, except in cases of HSP with delayed-onset purpura [6]. Here we describe a case of delayed-onset purpura with GI involvement in which high-dose intravenous methylprednisolone pulse therapy (IMPT) was effective.

## Case presentation

**Figure 1:**
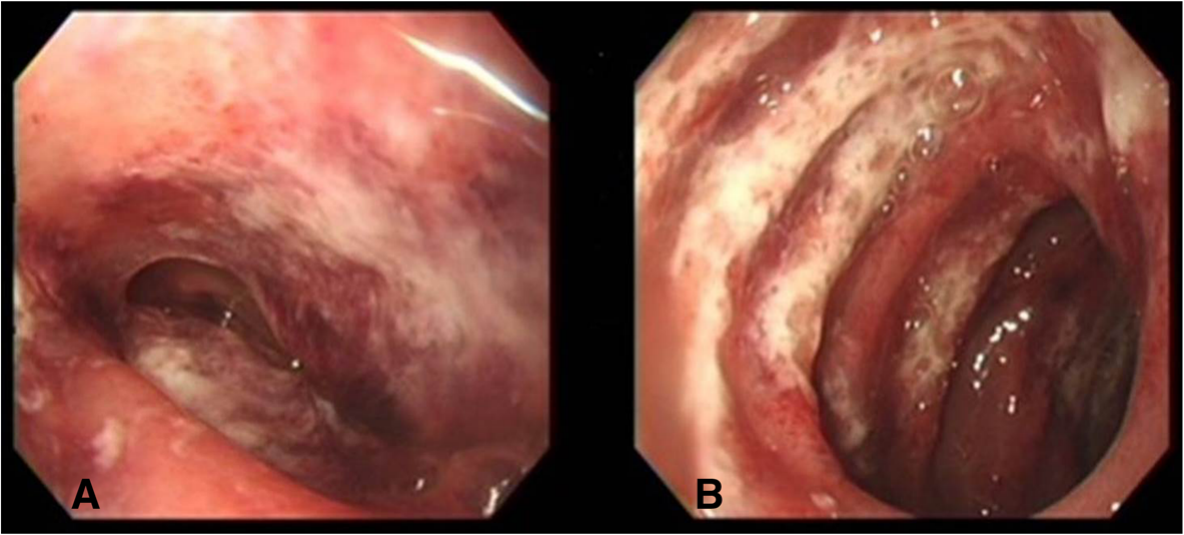
**Esophagogastroduodenoscopy of the Henoch–Schönlein purpura-affected bowel. A)** the antrum of the stomach and **B)** the duodenal second portion. This image shows the descending and transverse portion of the duodenum in the patient with hemorrhagic duodenitis and duodenal vasculitis with the additional finding of a purplish and reddish mucosal edema.

In July 2013, a previously healthy 15-year-old Korean girl presented with abdominal pain and bile-colored vomiting, which began 6 days prior. She had poor food intake since the onset of symptoms. Her urine output started decreasing daily before hospital transfer. She lost approximately 6kg in a week (from 46kg to 40kg). There was no sign of purpura. Her blood pressure (BP) was high (130/90mmHg), heart rate was 114 beats/minute, respiratory rate was 22 breaths/minute, and temperature was 37.5°C. However, laboratory and urine analysis results indicated no significant abnormalities, except 1+ proteinuria due to dehydration. On hospital day (HD) 1, an esophagogastroduodenoscopy (EGD) was performed, and diffuse superficial ulceration with whitish membrane and mucosal bleeding was noted in the descending and transverse portion of her duodenum (Figure 1). A purplish and reddish mucosal edema was also noted, indicating hemorrhagic duodenitis and duodenal vasculitis due to HSP. IMPT (60mg/day, 1mg/kg/day to a maximum of 60 to 80mg/day) and intravenous pantoprazole/ranitidine (gastric acid pump inhibiter/H_2_-blocker) were administered because of difficulties with oral intake. Intravenous pethidine and morphine were used for pain control but were ineffective.

On HD 4, a second EGD was performed because of worsening abdominal pain. The previous lesion had slightly improved. However, diffuse superficial ulceration with edematous and dark brownish mucosa was noted in the descending and transverse portions of her duodenum. Under the presumptive diagnosis of duodenal vasculitis caused by HSP, high-dose IVIg was administered (2g/kg/day).

**Figure 2:**
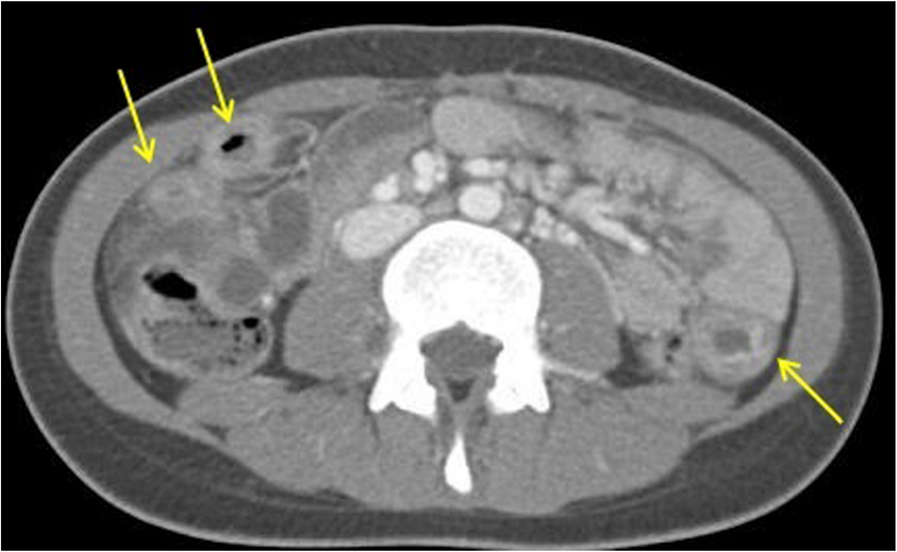
**Computed tomography of the abdomen showing an edematous bowel**. This image shows multifocal areas of bowel wall thickening, mesenteric edema, and vascular engorgement in jejuno-ileal loops (yellow arrows).

On HD 7, she experienced dyspnea and persistent abdominal pain. She had an acutely ill appearance. Her BP and temperature increased to 140/80mmHg and 38.3°C, respectively. Her serum was negative for antinuclear antibody and anti-neutrophil cytoplasmic antibody. Serum complements 3 and 4 (132mg/dL and 16mg/dL, respectively) were within normal limits. Serum IgG, IgA, and IgM levels were 3629mg/dL (normal range, 639 to 1349mg/dL), 162mg/dL (normal range, 70 to 312mg/dL), and 139mg/dL (normal range, 56 to 352mg/dL), respectively. Urine analysis revealed microscopic hematuria. An abdominal computed tomography (CT) scan showed an edematous bowel (Figure 2). Intravenous cefotaxime was added because of her persistent high fever and inflammation. IMPT (60mg/day) was discontinued because of pancreatitis.

**Figure 3:**
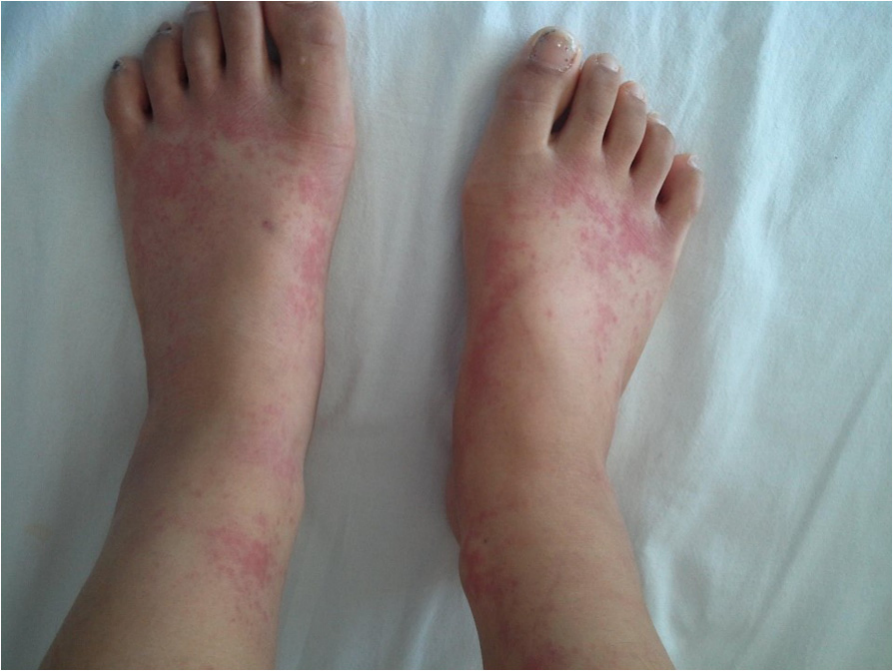
**Palpable purpura on both of the patient’s ankles and feet on hospital day 11.**

On HD 11, purpura developed on both of her ankles and feet (Figure 3). Her temperature was still high (38.2°C). A laboratory examination still showed an elevated white blood cell count (WBC; 22,200/μL) and erythrocyte sedimentation rate (ESR; 64mm/hour). Her amylase, lipase, and C-reactive protein (CRP) levels increased to 190IU/L, 210U/L, and 17.14mg/dL, respectively. Since pancreatitis had not improved after discontinuing IMPT, we readministered low-dose IMPT (0.5mg/kg/day).

On HD 14, her abdominal pain was aggravated, but the purpura disappeared. She still had a fever (38.0°C). Her laboratory results showed a much higher WBC (34,700/μL) and a declined but abnormal ESR (15mm/hour) and amylase (74IU/L), lipase (62U/L), and CRP (8.16mg/dL) levels. However, her random urine protein to creatinine ratio (UP/Cr) value of 0.41 had slightly increased. Because of her aggravated abdominal pain, an emergency abdominal CT scan was performed, showing more severe bowel edema. Consequently, the IMPT dose was increased to 1 to 2mg/kg/day, with the addition of intravenous metronidazole. On HD 17, the second IVIg was infused (2g/kg).

**Figure 4:**
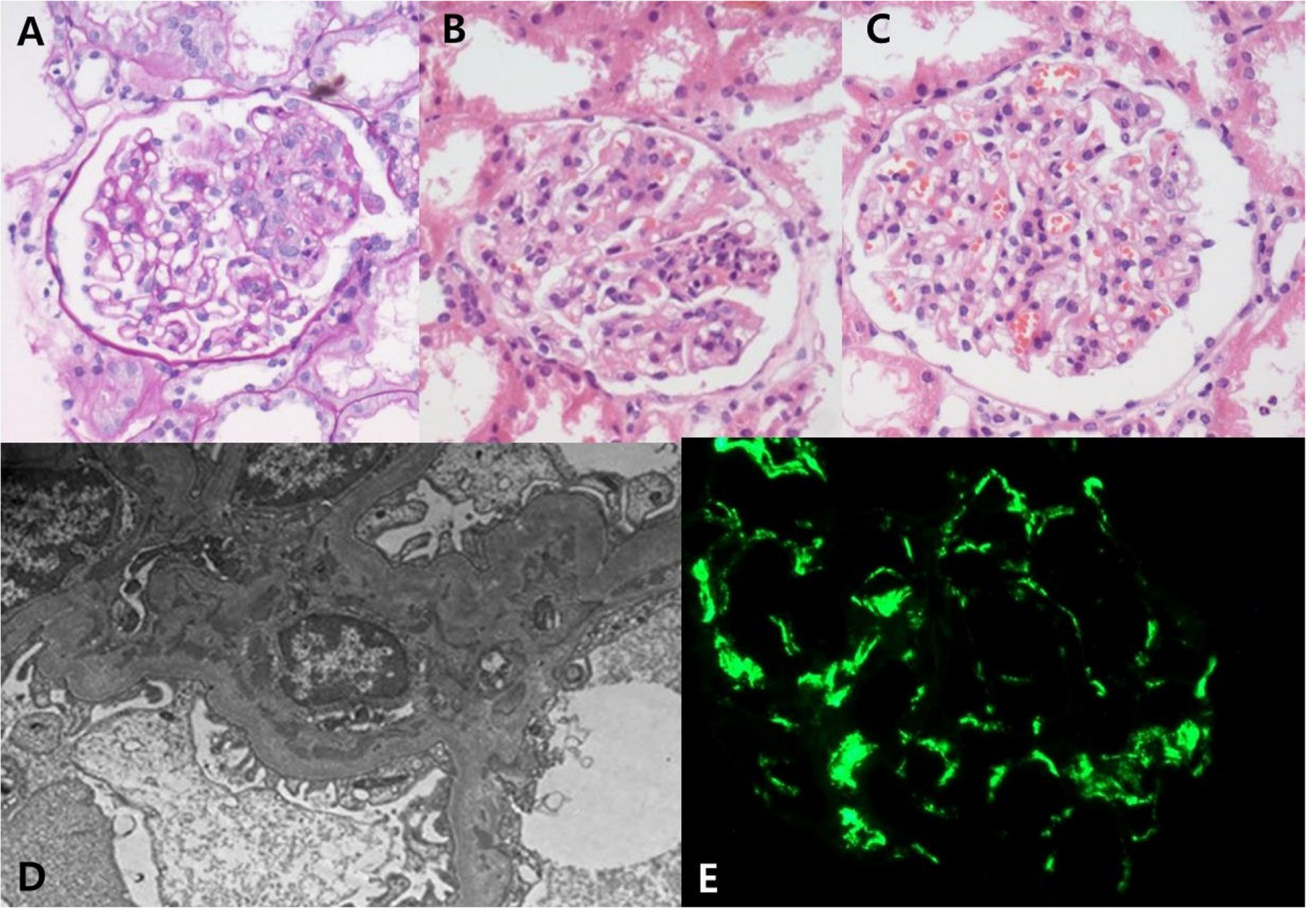
**Kidney involvement of Henoch–Schönlein purpura.** The biopsy reveals 9% segmental loop necrosis. **A)** Polymorphonuclear cell infiltration and **B)** segmental mesangial proliferation. **C)** Periodic acid–Schiff stain with hematoxylin and eosin stain, ×400. **D)** The mesangial deposit is observed on electron microscopy. **E)** The immunoglobulin A deposit is 3+ on immunofluorescence microscopy. Overall, grade II Henoch-Schönlein purpura nephritis is diagnosed based on the International Study of Kidney Disease in Children grading system [7].

On HD 20, her fever and abdominal pain persisted, and gross hematuria (GHU) developed. Laboratory results showed the following abnormalities: WBC count, 23,600/μL; ESR, 67mm/hour; amylase, 125IU/dL; lipase, 104U/L; and CRP, 6.41mg/dL. The random UP/Cr had elevated to 2.15. The antibiotic was switched to piperacillin and tazobactam to eradicate the infection. A kidney biopsy was performed. Finally, a decision was made to administer high-dose IMPT for 3 days consecutively. The renal biopsy showed an increased glomerulus size, segment hypercellularity, 9% segmental loop necrosis on light microscopy, mesangial deposits on electron microscopy, and IgA 3+ on immunofluorescence microscopy. Overall, the biopsy revealed grade II HSP nephritis according to the International Study of Kidney Disease in Children grading system [7], and HSP nephritis was confirmed (Figure 4).

After the course of high-dose IMPT, her abdominal pain and fever dramatically disappeared. On HD 30, an oral, not intravenous, steroid (1mg/kg/day to a maximum of 60 to 80mg/day) was administered, because the patient was able to eat. GHU and proteinuria persisted. Therefore, enalapril was prescribed for the proteinuria (5mg daily), and she was discharged. The oral steroid was discontinued after 6 months, and only enalapril was continued for an additional 2 months. Urine analysis results at the last follow-up in April 2014 were as follows: proteins, negative; red blood cells, 1 to 4/high-power field; and UP/Cr, 0.047.

## Discussion

The patient was admitted to our hospital with upper abdominal pain and bile-color vomiting. On admission, there was no evidence of intestinal obstruction or acute pancreatitis during the initial physical examination or on radiographs. Therefore, EGD was performed to determine if a gastric ulcer such as HSP duodenitis was present. Based on the EGD findings, duodenal vasculitis due to HSP was diagnosed. It is not rare for a patient with HSP to present with skin purpura prior to having abdominal symptoms. However, in our case, HSP was associated with delayed-onset purpura and GI involvement at disease onset. Approximately 14 to 36% of cases of HSP show GI involvement with delayed-onset of purpura [3]. Therefore, if a patient complains of severe upper epigastric pain, duodenitis due to HSP must be considered even though skin purpura may be absent.

Treatment for HSP is controversial. Although in some cases the symptoms may resolve without any therapy, corticosteroids and IVIg have been used in patients with HSP with severe GI involvement as standard and alternative therapy, respectively [4,5]. In this case, the patient complained of repeated severe abdominal pain accompanied by abdominal distention, dyspnea, fever, and pancreatitis. The symptoms did not resolve even after administering twice the amount of low-dose steroid treatments, followed by twice the amount of IVIg injections. Severe edema of her small intestine was also observed on CT. The efficacy of double IVIg injections in most cases of refractory intestinal involvement by HSP is unknown; however, it did not work in our case.

IMPT may aggravate pancreatitis. However, serial changes of amylase/lipase worsened even after discontinuing the steroid. Thus, pancreatitis was not induced by IMPT. Furthermore, in most cases, HSP would be accompanied by pancreatitis. We considered that pancreatitis was a complication of uncontrolled HSP.

Studies have reported that high-dose IMPT is rarely used to stop the progression of nephropathy in cases of HSP nephritis with a severe crescent on renal biopsy [8]. However, a few case reports have suggested high-dose IMPT for HSP with GI involvement [6]. In this case, renal biopsy was performed because of the GHU on HD 20. Even though the biopsy showed that renal involvement was not as serious as assumed by the severe abdominal symptoms, high-dose IMPT was performed because the two standard treatments failed. After administering high-dose IMPT, her fever continued for 2 weeks while severe abdominal pain and whole body discomfort disappeared. Since she was able to eat independently, intravenous nutrition was discontinued. Therefore, all of the abdominal symptoms recovered simultaneously with high-dose IMPT.

In our case, a high serum level of IgG was noted. However, it was assumed that this elevation was because IVIg was the previous initial treatment.

Besides, it is more important to recognize that severe abdominal symptoms (bloody stool) and persistent purpura for >1 month are risk factors for renal involvement [9]. In this case, GI symptoms were severe, with less involvement of the kidneys. When GI symptoms are severe, the possibility of another disease (for example, Crohn’s disease, ulcerative colitis, microscopic polyangiitis, or Wegener’s granulomatosis), which are capable of mimicking HSP, should be ruled out [10-13].

A low level of factor XIII activity correlates with the severity of HSP’s clinical manifestations, particularly abdominal pain and GI bleeding [14,15]. In our case, we could not quantitatively determine the factor XIII level. One study suggested that the administration of factor XIII may be useful in the rapid improvement of severe abdominal pain and GI bleeding [15].

## Conclusions

High-dose IMPT can be used as the ultimate treatment for delayed-onset HSP with severe abdominal pain when symptoms do not improve after administering low-dose steroid and IVIg treatments.

## Consent

Written informed consent was obtained from the patient’s father for the publication of this case report and any accompanying images. Upon request, a copy of the written consent is available for review by the Editor-in-Chief of this journal.

## Abbreviations

BP: Blood pressure
CRP: C-reactive protein
CT: Computed tomography
EGD: Esophagogastroduodenoscopy
ESR: Erythrocyte sedimentation rate
GHU: Gross hematuria
GI: Gastrointestinal
HD: Hospital day
HSP: Henoch–Schönlein purpura
Ig: Immunoglobulin
IMPT: Intravenous methylprednisolone pulse therapy
IVIg: Intravenous immunoglobulin
UP/Cr: Urine protein to creatinine ratio
WBC: White blood cell count
